# Annual Report of the Department of Health of Chicago, for 1884

**Published:** 1885-09

**Authors:** 


### DEPARTMENT OF HEALTH.

CHICAGO, APRIL 1ST, 1885.

TO THE HON. CARTER HARRISON, MAYOR, AND TO THE HONORABLE CITY COUNCIL:

I herewith transmit the annual report of the Department of Health of the City of Chicago for the year ending December 31st, 1884.

With this report is also published the statistical report for the year 1883. Such recommendations and modifications of ordinances as I have thought proper to bring before the City Council, during the past two years, for their action, have been sufficiently commented upon, while under discussion, and do not require further reference.

### CLEANING THE CITY.

The custom of assigning the cleaning of streets to the care of the Department of Public Works, and the cleaning of alleys and removal of house garbage from alleys and streets to the Department of Health, has resulted in the usual confusion and extravagance of expenditure, which have, heretofore, more or less, characterized the work.

The cleaning of a great city—and keeping it clean—is a labor of enormous magnitude and of the most vital importance, and as there is really no demarcation between filth of the street and filth in alleys, there should be no division of responsibility in its removal, and, in my opinion, one contract should cover the whole. Such a course *should* insure efficiency and economy.

INSPECTION OF FACTORIES, WORKSHOPS AND DWELLING HOUSES has become a very important work of the department, and our diminishing death-rate may be largely attributed to the active supervision and improvement of the houses and places of labor of our laboring classes. This supervision has covered about 263,000 of our population, and the statistics presented by the chief of that bureau will be found of interest. The force has, at this date (April 1st), been increased to eighteen men, and I anticipate much assistance from their services.

### STOCK YARDS AND MEAT INSPECTION.

This service, so important to the comfort and health of our citizens, has been satisfactorily performed. The stock-yard district has been the rendering neighborhood for a large portion of the civilized world. The fresh meats slaughtered there are distributed in carcass to all the larger cities of America, while many of the villages or small cities of 10,000 inhabitants or more of the New England and Middle States have refrigerators supplied from the same source.

Large orders have been filled by our packers for preserved meats in tin cans for the use of the English army in Egypt and India (21,559,548 ℔s. in year ending May, 1885), and these meats are found in every hamlet of Europe and America.

In 1884, there were slaughtered at these yards 1,025,813 beeves, 3,959,352 hogs, 511,278 sheep; and the blood, bones, feet, scrap and offal of these animals were rendered into grease, glue and fertilizers in so prompt and efficient a manner as to create no offense, unless caused by unavoidable accident to machinery or lack of water supply. Officers of this department are on duty day and night at the rendering houses.

The inspection by meat-inspectors of the department, of animals brought to the yards and designed for food, has given rise to considerable conflict with owners or commission men, to whom the animals were consigned.

The reputation of the business is so vitally connected with a careful inspection, that the stock-yard authorities and large packers have always sustained the health-officers in a vigorous performance of their duty. All animals regarded as unfit for food are shot in the pens and turned over to the rendering company. Several suits against officers of the department for damages, for loss of stock, have been brought during the year, but abandoned when they reached the higher courts.

On the 28th and 29th of July, two shipments of stock—comprising 964 head—reached the yards, afflicted with the southern cattle fever. Seven hundred and twenty-five were immediately shot and delivered to the rendering tanks. The remaining animals—apparently in good health—were slaughtered, under the inspection of Mr. Lamb, and about forty per cent. of them disclosed evidence of disease, and the carcasses were condemned. The animals had been grazed all summer, along the northern line of the Indian Territory, and were driven to Caldwell, Kas., placed on board cars and taken to Kansas City, Mo. On the day of their arrival at Kansas City, several of them died, and the stock-men becoming alarmed rushed them off to Chicago.

It is presumed that this experiment, which cost the owners $16,000, will convince them that Chicago is not the most desirable market for stock of this character.

### THE RIVER.

The condition of the river has been so satisfactory as to give little cause for complaint. The south fork of the south branch still remains in a deplorable condition. I can only repeat what I had to say in my last report on this point: "There can be no question that this slough of filth coursing through the southwestern portion of our city, is a terrible menace to the health of our citizens. It not only directly creates disease, but by the septic influence it generates, invites the location and development of such germs of epidemic disease as may be introduced from without."

GENERAL HEALTH.

There is no feature of the Registrar's report which calls for comment. The city has been free from epidemics of every nature, and the death-rate is remarkably low.

OSCAR C. DEWOLF, M. D.,
*Commissioner of Health.*

---

### BUREAU
### OF
### TENEMENT AND FACTORY INSPECTION.

---

OSCAR C. DEWOLF, M. D.,
*Commissioner of Health:*

SIR:—I herewith present a report of the work performed by the tenement and factory inspectors during the year 1883.

Sec. 1352 of the Municipal Code requires a full and detailed statistical report of the number of males and females of all ages employed in labor or service in factories, workshops, stores, warehouses, elevators, yards and domestic workrooms.

There are about 19,000 of such above named places of employment enumerated in the subjoined tabular statement, and about 200,000 persons employed therein.

The number of adults at the age of working capacity is much greater in proportion to the whole number of inabitants in this city, which is being constantly crowded with immigrants, than in older cities having a native-born settlement of Anglo-Americans, whose enterprising young people emigrate to all parts of the continent in search of more remunerative employment.

The principal business centre of the south division of the city has lately been bounded and confined at Polk street through the location of a railroad terminus ; and the Board of Trade building has been erected at the foot of LaSalle street, between Jackson and Van Buren streets.

This encourages builders to erect ten and twelve story blocks for offices, workshops, and living apartments, so as to derive the largest obtainable profits from their investment in building grounds.

One such turret building will accommodate as many persons of employment (over and on top of each other) as an entire block of low business buildings outside the circumscribed business centre.

The detailed reports show that *more* than 60,000 persons are employed at service or labor *above* the first or store floors in the first ward alone (the business centre); the upper floors constituting one vast workshop.

Among the noticeable conditions found in the high buildings, and which are not sufficiently regulated by ordinance, is the lack of proper safe-guards in case of fire or accidents.

These high structures, constantly crowded as they are with human occupants, intermingled with highly inflammable goods, should consist entirely of fire-proof materials, and should be supplied with fire-proof outside stairways, constructed so as to afford safe exits to the vast numbers of architects, printers, telegraphers, cloak, clothing and suit makers, and the multitude of others who are employed in the several professional, commercial or manufacturing pursuits.

The prevailing custom of providing comfortable passenger elevators, together with the cheaper rents and better light and air to be obtained, causes the upper stories of these tall buildings to be much sought for, thus supplying one of the principal inducements to capitalists to erect an increased number of such buildings each year.

However, the convenient elevator has reduced the number of necessary stairways, constructed as they are for *general* use, and with little or no precautions for accidents, panic or fire.

These elevators should be constructed of incombustible material and enclosed within a fire-proof shaft; but this is rarely done—the usual method being, to but partially enclose them with a light wire-netting, or other similar materials, thus providing a sure passage for dense smoke or fire, and rendering this elegant convenience entirely useless at the very moment it is most needed, as a means of escape.

The lower or ground floors only, are used as stores and to show sample goods; all upper floors are workshops, if not offices or furnished rooms.

The style of fire-escape generally used at present is primarily intended for the use of the firemen, who can climb up and connect and direct the hose for a stream of water to the several upper floors; but the employés could not use these perpendicular ladders to safely escape from the twelfth story of a burning building, unless they are professional acrobats.

Iron stairways should be provided within fire-proof enclosures (which could be properly lighted through mica-covered openings), to connect at each floor with suitable *outside* iron balconies, in connection with outside iron stairways, and all provided with suitable railings, etc.

The ordinance prohibiting the employment of children under fifteen years of age, for more than eight hours each day in factories and workshops, is being very generally complied with.

The classified, alphabetical lists of the trades and occupations are made according to the titles of the employing firms, and not by the articles of manufacture or sale; a more detailed or substantial classification being deemed impracticable, because of the almost universal custom of firms and companies of concentrating several trades and related occupations into one establishment, as printing, binding, lithographing, stationery and general publishing.

The subjoined alphabetical tables fully explain themselves and show an activity and magnitude in the manufacturing industries of this city, not so accurately obtainable from any other known source.

### TENEMENT HOUSE INSPECTION.

A thorough examination was made during the year of all the sanitary conditions in 3,744 tenement houses, which contained 9,164 families, consisting of 42,331 persons.

A complete detailed written report is filed in this department for *each* examination made, the action taken, if found in an unsanitary condition, and the improvements effected. From the general character and conditions found in these buildings, filled in many instances with noxious odors from defective drainage, etc., and to twice their statutory capacity, with a class of inhabitants who are not over-clean in their personal habits, it would seem to be almost necessary that a still closer supervision should be exercised over this class of work.

I would suggest that a careful and thorough house-to-house examination be made of all the tenement houses within the city, during the months of April, May and June, of *each* year, in order that the neglected repairs and accumulations of the winter months may be rapidly and effectually made or removed and a considerable cause for sickness, and possible deaths, done away with. The owners should be compelled to provide suitable and permanent receptacles for the garbage and general refuse of these houses, after which the inspectors should see to it that the occupants make *use* of these receptacles for their intended purposes; also, that the day scavengers remove the contents promptly and regularly. This would require constant and rather unsatisfactory work (for the inspectors) for a few months, but the resulting benefits to the tenants, if not to the general public, would fully justify the necessary expense and trouble; as, no doubt, the occupants would soon adopt the more cleanly habits because of their enhanced comfort, and in a short time follow the suggestions of the inspectors from *choice* and not by compulsion.

In the year 1883, (only two years since,) a comparative statement was compiled in this bureau, of the mortality ratio per thousand inhabitants in the strictly private residence wards and those containing tenement-houses and inferior dwellings. These comparisons were taken from the "record of vital statistics" of this city, and are therefore reasonably accurate and to be relied upon as correct, and the results show that nearly *three* persons die in the tenement wards for every *one* person dying in the residence wards, and making a further comparison with the *class* of disease (preventable) causing death, the ratio is much greater as against the unsanitary wards. We will not claim that *all* the excess in the mortality is due to the unsanitary *housing* of these people, but conceding that *one-half* is due to insufficient and unwholesome food and excessive manual labor, and we still feel that additional work in this direction may be prosecuted with beneficial results. In the 3,744 tenement houses examined, the inspectors found it necessary to serve 921 written notices to make *permanent* sanitary improvements in the lighting, ventilating, plumbing and drainage of the buildings. In addition to the work mentioned and shown in the detailed reports, a complete record is made of the number of rooms, families, persons, males, females and children occupying *each* house, which would be of great value and use, should an epidemic spread through the city.

SPECIAL EXAMINATIONS.

There were 1,558 "special" examinations made in residences or dwellings containing one family each, all of which were made upon the written requests of physicians, occupants or owners, or both. This class of work was not at first intended to form a part of the "regular" work of the inspectors; but the public generally are recognizing the true value and benefit to health of perfect house sanitation, and making such rapid strides in voluntary self-education in this direction that it has been deemed consistent to accede to those requests, and suggest proper remedies for all defects found.

It would seem that this "special" work was not the least important one of this department, when it was found that, out of the 1,558 houses examined, 726 were deficient in one or more of their important sanitary conditions, which defects were immediately remedied under the supervision of the inspectors.

At first thought, these facts would lead one to believe that *all* places of habitation should have the same inspection, *regularly* made, as are in the tenement houses; but the facts are, that these "special" examinations are only made upon the written requests of a physician (professionally visiting the premises named), or from the occupant or owner, who has long endured an indescribable odor (generally emanating from defective drainage or plumbing) throughout the house; and, as a last resort, seeks the health department, that they may be relieved of the cause of their troubles.

### SANITARY REQUIREMENTS IN NEW BUILDINGS.

The laws of this state require that the plans and specifications for all places of habitation (in cities of 50,000 inhabitants), shall be submitted to the Commissioner of Health for his approval or rejection, so far as relates to all sanitary arrangements to be provided in said buildings, and includes the ventilation of rooms, light and air shafts, windows, ventilation of water-closets, drainage, plumbing, etc.

Permits have been issued during the year for 2,444 buildings for habitation; the plans for which were submitted to this office for approval, so far as relates to *all* the sanitary arrangements to be placed in the proposed buildings, and copies of 1,389 of said plans were properly filed for reference, and represent all classes of house building, from the smallest cottage house to the palatial residence, and from the ordinary or usual two-flat building to the towering seven-story tenement house.

Of the 2,444 plans submitted, 1,142 were for so-called "flat" buildings (buildings fitted for *one* family only to each story or floor), 294 of which contained stores on first or ground floor; 483 tenement houses, containing *three* (3) or *more* families each, were built, and one, now in course of construction, to contain thirty-four (34) families; 116 cottages (brick) were built to accommodate one (1) family each, and the remaining 702 houses include all classes of one and two family buildings, from the mansion of the millionaire to the hostler's living apartments in the upper story of a barn.

These houses were constructed for the accomodation of 4,551 families, and estimating that each family consists of four persons, we have a total of 18,204 persons, who have received a direct and *permanent* benefit through the exertions of this department. The method pursued to accomplish these permanent benefits or improvements consists in first compelling all architects, builders and other persons interested in the construction of these buildings (and before commencing work on same), to first submit their plans and specifications to the Commissioner of Health, for his approval or modification as to their sanitary arrangements; and after approval, this bureau enforces all the requirements of the laws, ordinances, rules and regulations relating thereto, by constant and supervisory examinations of the buildings from time to time, during their erection and after completion.

This supervision is found necessary, because many unprincipled persons often attempt to omit necessary conditions or to construct in a much different manner than the approved plans and specifications represent, and in direct violation of the laws, etc., governing the same. The total number of these violations by the architects, builders and plumbers in the *construction* of new habitations during the year were 204, which required the serving of a written notice by this department (for each one), requesting the violators to remedy the work at once, which order or notice not being fully complied with, the whole was carried into the courts where, I am pleased to state, *every case* (and there are scores of them) was decided in favor of this department. Therefore, without *legal* compulsion, through the efforts of competent inspectors, these sanitary improvements could not have been enforced, and the construction of permanently unsanitary house-buildings would have continued as in the past. The importance of, if not the *necessity* for, this class of sanitary work, cannot be better shown than in the preceding statement, that in *one* year alone this bureau has effected permanent and substantial improvements, which directly benefit *more* than eighteen thousand inhabitants; while under the former ordinances these improvements could only have been enforced *after* the buildings were occupied, and then only partially and in an imperfect manner.

As will be seen, nearly one-half of all the houses constructed were of the class known as "flats," and with very few exceptions are rented for $20 to $125 per month for *each* flat or family, a sum beyond the ability to pay, of the clerks and so-called middle class of merchants, employés and mechanics in our city.

This brings us face to face with the unpleasant fact, (as mentioned in my last annual report,) that practically *no* provisions are being made to house the *toiling* multitude of wage-workers in our city, and in order that a clerk or other small-salaried man may procure suitable rooms for the proper accomodation of his growing family, he is compelled to travel far toward the city's suburbs and suffer the inconveniences of spending hours each in day going to and from his place of employment in addition to a considerable expenditure of cash. The laboring classes employed about our docks, stores and warehouses in the central portion of the city cannot go to the suburbs, nor any locality of any considerable distance from their places of employment, because of their salary being so small as to preclude them from paying any portion of it for transportation, and so it follows that they are compelled to remain in the down-town overcrowded and poorly-lighted tenement quarters, with immoral surroundings and too generally poor school accomodations for their children. It cannot be questioned but that buildings of four or more stories in height could be erected on cross streets in close proximity to the business centers and arranged in small but light, airy and convenient apartments, most thoroughly provided in a sanitary way, and rented in suites, for 8 to 12 dollars per month for each family, and which would return a *net* percentage on the investment far above an equal investment in other classes of houses in any portion of the city distant from the trade centers.

If it be possible to build with great profit and *in the most scientific manner*, whole cities like Saltaire and Pullman for the *exclusive* housing of operatives (employés) of large manufacturing and commercial institutions, then why is it not only possible, but highly profitable, and withal a very philanthropic enterprise to erect blocks of tenement houses on the most approved plans for the wage-working poor in this city, and thereby obliterate such localities as the "Patch" and "Cheyenne?" Such an enterprise would abolish these rookeries by causing them to be unsought for as places of habitation; it would improve the morals of our lower classes by improving their surroundings and education and largely reduce the mortality among this class of our populace.

LODGING HOUSES.

The improvement of the so-called lodging-houses in many of the basement or cellar stories along a few of the central streets has been the cause of much labor on the part of this department in forcing the owners to keep them in a fair sanitary condition and prevent excessive overcrowding.

Not a few have have been declared uninhabitable and the owners compelled to permanently vacate them and estopped from further using them for such purposes.

In other instances the entire bedding has been burned or otherwise destroyed and replaced with new, but a less number of beds. However, real, genuine *satisfactory* results cannot be obtained in this class of work until the city ordinances are so amended as to entirely prohibit any person from sleeping or dwelling in any basement or cellar *under any circumstances whatever*.

Respectfully,

W. H. GENUNG, *Chief Inspector*.

### BUREAU
### OF
### TENEMENT AND FACTORY INSPECTION.

---

OSCAR C. DEWOLF, M. D.,

*Commissioner of Health:*

SIR:—I herewith present a report of the work performed by the "Tenement and Factory" inspectors for the year 1884, including about all classes of sanitary work of the health department as required by the following ordinance, except the scavenger service and the care of contagious diseases.

Section 686 of the city ordinances declares that, "It shall be the duty of the commissioner of health to enforce *all* the laws of the state and ordinances of the city in relation to the sanitary regulations of the city, and cause all nuisances to be abated with all reasonable promptness, etc., etc.

This ordinance is very general in its character, but there are many others which apply directly to specified unsanitary conditions in all classes of buildings; however these ordinances should be so amended as to provide for abatements in a special manner. This annual report shows a division of the sanitary work into three separate parts, viz:

First. The sanitary work performed in *occupied* places of habitation and which includes *all* buildings wherein any person may dwell or lodge.

Second. The control of the sanitary conditions and safety as relates to egress, protecting machinery and storage of dangerous materials in places of employment or service.

Third. The exclusive control under the state laws of all the sanitary arrangements or conditions, such as the heating, lighting, ventilating, plumbing and drainage to be provided in every building within the city, during its construction and which is to be used as a place of habitation.

Section 1347, of the city ordinances, declares "that no person shall hereafter erect, or cause to be erected, or converted to a new purpose by alteration, any building or structure which, or any part of which, shall be inadequate or defective in respect to ventilation, light, sewerage or any of the usual, proper or necessary provisions or precautions for the preservation of health."

The state laws invest the Commissioner of Health with authority to control all the sanitary arrangements to be provided in any habitable building within the city.

The enforcement of these laws by the inspectors has been the means of accomplishing more valuable sanitary work than by all other ordinances combined, in that by their enforcement all improvements made are of a *permanent character*, and place the building (except in cases of accident) in a permanently good sanitary condition, thereby benefiting all occupants uniformly throughout the city.

The construction of dark, damp, unventilated living rooms has been wholly prohibited for the past two years. All water-closet rooms are provided with sufficient external light and ventilation and which are never permitted to be in any way directly connected with any habitable room. In fact, all sanitary conditions are enforced which are conducive to the health of the occupants.

During the past year much work has been accomplished in greatly improving the sanitary conditions in a number of our public school buildings, which in some instances amounted to an entire remodeling of the drainage systems and the heating and ventilating apparatus.

One school building was torn down, as recommended by this department, to be replaced by a larger and more modern structure. This work will be vigorously prosecuted, with the hope of placing *all* the school buildings in the best possible sanitary condition during the present year.

As will be seen from the following tabulated reports, the inspectors have, during the year, made a total of 28,092 examinations in places of habitation and employment, and filled proper detailed written reports for each building and firm. These examinations are exclusive of the work performed under the state laws relating to the sanitary supervision of 3,240 new buildings in course of construction for habitation, correct plans for 1,941 of which have been filed with this department for future reference, but which cannot appear in this report in detail because of the voluminous form of such reports. The owners or occupants of 1,932 houses of all sizes, each containing from one to twenty families, also employés of 483 shops and stores complained during the year of insanitary conditions in the plumbing, drainage, ventilation, etc., existing in said buildings, for each of which a special examination was made by an inspector and the defects properly corrected.

But this number of complaints does not cover half the work of house-inspection, there having been 4,394 houses containing 10,445 families reported in detail, besides the great number of cottages and dwellings which were greatly improved in their permanent sanitary arrangements through verbal notices or suggestions of the inspectors.

The sanitary inspections are made from house to house, but the record of special reports *in detail* covers only the places of habitation found in bad sanitary condition and otherwise contrary to the health-regulations and for the abatement of which a written notice was served. Whenever a sufficient number of officers (inspectors) can be detailed to examine into the sanitary condition of *all* buildings, then a complete street-list, with every building used for habitation or employment, will be placed on record, in order to show the sanitary arrangements of each and all of them, together with the number, sex, age and nationality of the occupants.

Many portions of the city are in a poor sanitary condition from lack of proper drainage, and this department caused the laying of new street sewers in certain crowded districts of the 5th, 6th and 14th wards ; also compelling the property owners to make proper sewer connections with their houses. In one district in the vicinity of the lime kilns in the southwestern portion of the city, a population of 6,500 persons was benefitted through such improvements.

18,438 places of employment (factories, stores, etc.,) were visited and detailed statistical written reports filed as required by law.

The employment of children under fifteen (15) years of age for more than eight hours a day is forbidden by ordinance, and the employers in factories comply with this requirement generally throughout the city. Improvements of a permanent character were effected in 4,229 buildings by all classes, and included the construction, repairs and cleaning of catch-basins, trapping and ventilating of house-drains, sewers, soil and waste pipes, ventilating bed rooms, work rooms, bath and water-closet rooms, supplying light and ventilating shafts, providing adequate water supply to tenements and factories, supplying proper "traps" for plumbing and drain pipes, guarding dangerous machinery, providing proper egress for factory employés, removing dangerous material from rooms occupied by factory employés, and many others of a miscellaneous character, but not specially named in the inspectors' reports. Very many more improvements were effected through verbal suggestions of the inspectors and which were not placed on the books of record.

A considerable number of suits were brought against violators for non-compliance with the written notices served by inspectors, but the *ratio* of these suits is decreasing each year.

However satisfactory this work may have been (and the constant decrease in the mortality rate is one proof of the satisfactory results) there is yet much to be done, when it is known that there are 70,000 dwellings [U. S. census of 1880 reports 61,069], and more than 25,000 places of employment, all of which should be regularly examined at stated intervals, as the law provides, by a competent officer of this department who should compel the proper persons to immediately remedy all insanitary defects.

The most varied and complicated work of the inspectors is in the dwellings and tenement houses, more than four thousand of which have been examined and much improved during the year. Nearly all of these houses were located in the so-called "patches" and required the constant attention of all the inspectors for the greater part of the year. As there is about eleven thousand tenement houses in the city, it will readily be seen that there should be provided at least one inspector for each ward, in order to carry out the provisions of the ordinances throughout the city, which covers an area of 36 square miles and contains a population of 650,000, with a yearly increase of 30,000.

Section 1352 of the municipal code requires a record to be kept, and an annual report to be made to the City Council, which shall specify:

1. Number of males and females of all ages employed, also number of boys and girls under fifteen years of age employed.

2. The number of violations of this ordinance regulating the sanitary supervision of all places of habitation and employment, and the number ot abatements, with detailed account of improvements effected.

3. General and special sanitary condition of all people in labor or service in factories, workshops, stores warehouses, elevators, yards and domestic work-rooms.

4. Number and kind of dangerous and unhealthy employments, and diseases of the several trades and occupations.

5. Statistics of labor, wages and cost of living, in connection with the several trades and occupations specified in the reports of the factory and tenement house inspectors.

Such reports shall be printed as public documents for the information of the people.

This requires the work of a competent recorder of statistics to classify all these reports, notices, and suits entered by the inspectors, and enter them in proper books of record, also to make weekly and quarterly reports, etc.

In the greater number of buildings erected as habitations for the working classes, the rooms are generally much too small, and the City Council should limit, by ordinance, the size of living and sleeping rooms as to cubic air-space and floor space for each occupant, to guard against preventable sickness from over-crowding.

Thousands of frame houses of all sizes have, in course of time, become untenantable through sagging of sills, doors, window frames, and other parts, also rotting of wooden floor-timbers, placed too near the ground, fouling of wooden drains and other causes, exhausting the vitality of the occupants by exposure to the elements at all times in a manner which, in many instances, is far more debilitating on the human system than the work which affords a living. We have no ordinance compelling the thorough repairing of these old rookeries, shanties and dwellings, so as to prevent the draught through the countless openings made by broken plaster, floors, window glass and "improperly fitted doors and windows," and an ordinance covering this defect should be enacted without unnecessary delay.

The *Act* of May 30th, 1881, for the "Regulation and inspection of tenement and lodging houses and other places of habitation, declares in section 3: that, "it shall be unlawful for any plumber or other person or persons to cover up or in any way conceal such plumbing work in or about such building or buildings until the Health Commissioner approves of the same," and makes the penalty for violation a fine of from one to two thousand dollars for each offense. There is not a shadow of doubt but that this *Act* has been of almost inestimable value to this department in *preventing* the construction of permanent insanitary conditions, and also the means of improving the standard of the plumbing work of this city fully one hundred per cent.; but this *Act* should be so amended as to compel all plumbing to be placed, whenever practicable, in sight at all times and always easily accessible. This would encourage the plumbers to perform better work and to use better material; it would also educate the general public to know good work and material when seen; it would greatly improve the sanitary condition of the habitations, it would facilitate repairs and save much vexatious delay in locating leaks, stoppages, and many damaging defects in the prevailing present method of concealed and mysterious plumbing. At the date of the passage of this *Act*, it was the prevailing habit among many plumbers to conceal a waste or service pipe whenever it was possible to do so, thus offering an opportunity to apply almost worthless work, and still worse material, and providing, in course of time, a sure means of seriously damaging the building in which it was placed, and making of the whole a complete mystery to any except the person who planned and executed the plumbing work. And this was done, first, because the owner desired to have the work performed as cheaply as possible, meaning perhaps for as little money as possible for what *he* deemed good work, said owner not having a knowledge of *good* work when seen or described; also because the compepetition among the contracting plumbers was so vigorous as to *seem* to make it necessary to apply the very cheapest material and workmanship in order to leave a living margin for themselves.

Section 2 of this Act provides that, "it shall be the duty of any plumber * * to receive instructions from the Health Commissioner before commencing the work in said buildings, etc., but these instructions relate only to the *method* of doing the work. This section should be so amended as to regulate the *material* used, at least to the extent that it shall be sufficient in size, strength and quality.

The state law or city ordinances or both should be so amended as to provide for metal sewer or drain-pipes for conducting the usual sewage of all places of habitation to some point outside the building walls; said drain-pipes to be in full view at all times, and in no instance to be buried in the earth or in any way concealed or covered up.

In cases where a cellar or excavation of any kind shall be deemed desirable, it would necessitate a double system of drains for sub-soil and storm-water drainage, both of which could be properly connected at some point outside the building walls. However, if the metal system had proper capacity (which would greatly increase the cost) the surface and storm-water and usual sewage of premises could be discharged into street sewer through this system, and the sub-soil drains could discharge into properly constructed gravel or sand wells or trenches. Too little attention is given to sub-soil drainage in large cities, where the earth is necessarily impregnated with filth, dangerous to health.

I believe that next to the four outside walls of a building the drainage is the most important, for without proper drainage any city building is practically unfit for human habitation. The ordinance relating to drainage should be so specific as to provide for not only the natural sewage of a building and the storm-water falling upon it and the connecting grounds, but should also provide for the sub-soil draining of the entire lot covered by the building to a depth of two or more feet below the foundation walls. Proper provision should be included to prevent the construction of walls in such manner as to become damp from the foundation upwards. Damp air and ground air should never enter a building to fill it with a disagreeable stuffy odor, which is alike unpleasant and dangerous to the health of the occupants. Another and not the least defect in the construction of house-drainage in this city is the universal arrangement of constructing one or more grease-receiving basins for each building.

These are usually constructed of brick in a careless manner, and rarely if ever water-tight, thus permitting the surrounding earth to become thoroughly saturated with the vilest filth. I can see no good reason for not abolishing these cesspools, which require constant attention and care to remove and bury their accumulating filth, thereby causing a very disagreeable if not dangerous stench for many hours during removal, or if permitted to over-accumulate, the drain becomes choked and the entire sewerage of the premises is necessarily discharged on the surface of the ground *under the building.*

This improvement, if made compulsory by law, would very much improve the general sanitary condition of habitations almost if not quite equally with the abolishment of the construction of privy vaults.

The sewage from a building of any kind, and especially a place of habitation, should be quickly removed from the premises to a point beyond the drain trap and into the street sewer, to prevent dangerous gases being generated from decomposing matter found in all drains. Any substance permitted to enter a private sewer or drain should never be retained in a "catch-basin" or cesspool a long enough time to allow it to become a mass of putrid decomposition, for this is the principal creator of sewer gas in houses. There is but one proper way to prevent this; that is, to prevent the accumulation, by prohibiting the construction of such cesspools, each with a capacity of several hundred pounds of filth.

Much thought and attention has been given to the proper housing of the renting public and more especially the working classes, such as clerks, book-keepers, mechanics, etc. The great expense precludes the erection of *small* tenement quarters provided with modern conveniences and thorough, necessary sanitary arrangements, except in large blocks.

And for many other reasons this would seem to be at least a partial solution of the problem of "what can best be done to provide proper homes for the renters?"

The practical application of this theory would appear in the plan of a model tenement block, similar to the following:

Taking a piece of ground, say 125x125 feet, running to an alley, there could be erected thereon a large fire-proof building in the form of a hollow square, and composed of a basement or cellar for storage purposes and heating and laundry apparatus; next, a high story for stores, shops, etc., with dwelling apartments in rear; then four or more stories to be used exclusively for tenement apartments, the top or attic story to be used for storage and drying rooms, or play rooms for children if desired. Such a building could be constructed in such a manner as to provide external light and ventilation for each and every room. The apartments to be so divided as to provide from two to four rooms with necessary closets for each family.

The water-closets could be erected in tiers (4 in number), one over another, with external light, and an adequate shaft artificially ventilated. A strong but airy iron balcony could encircle the large court at each floor, with connecting iron stairs for escape from fires or other accidents.

Suitable and sufficient elevators could be provided for use of occupants and for elevating fuel, etc. A janitor-engineer would have entire care and control of the entire building, to see that all its several mechanical parts were kept in perfect working order.

A building of this character, as compared with the present three-story tenement house, would produce a revenue in excess of the old style building exactly in amount to all rents received *above* the third story, and all these fourth and higher stories occupy the *same* ground as the lower ones, and therefore cost nothing for that item of expense.

By the use of elevators, the upper apartments are quite as accessible as the lower ones, and the building being fire-proof, makes them quite as safe and certainly more desirable and pleasant, because of better light and air, and being far removed from the dust, noise and bustle of the adjoining street.

In the present style of tenement building without a janitor's care, much trouble is caused to owners and occupants through careless and malicious actions of an unsanitary character; but which would be obviated in a large tenement block where *all* arrangements in common use by tenants are under the supervision of a competent person responsible to the owner, which owner is responsible to the health authorities. Such tenement blocks should be erected in or within easy distance of the commercial and manufacturing centres, to save expense of transportation to the employés, and also the time of going and coming to and from their places of employment; also that his family may have proper police protection and live within a reasonable distance from schools.

The moral benefits derived from the construction of such tenement blocks, with the privacy they afford to each and every family, can hardly be over-estimated, when taken into connection with the more cleanly personal habits to be obtained under these healthful surroundings.

The sense of shame becomes blunted in overcrowded, filthy, and ill-arranged apartments, and children become exposed to bad examples, which are apparently easier to follow than good precepts. It would seem proper that our large employers of labor should start this new departure in building tenement blocks for their multitudes of employés who would gladly remain their tenants.

The builders of these large blocks would greatly benefit the community at large by compelling the owners of inferior buildings to improve them to a proper standard of comfort and accommodation.

Respectfully,

W. H. GENUNG,
*Chief Inspector.*

### REPORT

OF THE

### REGISTRAR OF VITAL STATISTICS.

---

TO OSCAR C. DEWOLF, M. D.,

*Commissioner of Health :*

SIR :—I have the honor to present herewith the following annual report for the year ending December 31, 1883.

There ocurred during the year 11,555 deaths—a weekly average of 222. Of this number there were born in Chicago 6,257, and 1,554 elsewhere in the United States. There were 1,423 natives of Germany, 900 of Ireland, 150 of Canada, and 191 of England; 993 were natives of other foreign countries. One was born on the Atlantic Ocean, and one on the Mediterranean Sea, and the nativity of 85 was unknown.

Of the decedents, 3,850 were infants under one year of age, and 5,875 were children under five years of age, making 50.84 per cent. of the total mortality under five years of age. There were 1,121 over sixty years of age, including one over one hundred.

The death rate for the year was 19.26 per 1,000 inhabitants, estimating the population at 600,000.

Respectfully submitted,

M. K. GLEASON, M. D.,

*Registrar.*

### MORTALITY REPORT

### OF THE CITY OF CHICAGO

### FOR THE YEAR ENDING DECEMBER 31ST, 1884,

---

To OSCAR C. DEWOLF, M. D.,

*Commissioner of Health.*

There occurred during the year 12,471 deaths—a weekly average of 240, and during the preceding year 11,555, a weekly average of 222.

Of this number, in 1884, there were born in Chicago 6,997, and 1,537 elsewhere in the United States. There were 1,495 natives of Germany, 922 of Ireland, 157 of Canada, and 217 of England ; 1,069 were natives of other foreign countries. One was born on the Atlantic Ocean ; and the nativity of 76 was unknown or not given in the certificates of death.

Of the decedents, 4,179 were infants under one year of age, and 6,666 were children under five years of age ; making 53.45 per cent. of the total mortality under five years of age. There were 1,208 over sixty years of age, including two over one hundred.

The death rate of 1884 was 19.80 per 1,000 inhabitants, on a basis of 630,000 population, according to the school census taken in July of the same year. The death rate of the preceding year was 19.26 per 1,000 inhabitants, estimating the population at 600,000. The average death rate for the last five years was 21.96 against 18.51 for the preceding quinquennial period.

For further detailed statistics relating to causes of death, by wards and months, ages, nativities, sex, color, still-births, and social conditions, reference is made to the accompanying thirty pages of the report.

The best criterion of the sanitary condition of a community is best ascertained by showing the percentage of zymotic diseases and the number of deaths under five years of age, compared with the total mortality.

There were 4,216 deaths from zymotic diseases in 1884, making 33.81 per cent. of the total mortality for that year, against 3,666 deaths from similar causes, and a percentage of 31.73 for the year 1883.

In 1884, the greatest mortality from these causes occurred in the Sixth Ward, being 665 or 12.12 deaths to 1,000 population against seventy-three in the Eighteenth Ward (where the least mortality took place), with only 2.68 deaths to every one thousand of its population. Again, in the Sixth Ward, the percentage of the preventable diseases amounts to 42.82 of the total mortality, whilst that of the Eighteenth Ward is only 27.34 per cent.

In 1884, there were 1,111 deaths of children under five years of age in the Sixth Ward, making 71.54 per cent. of the total mortality, whilst in the Eighteenth Ward the percentage amounts to only 44.57.

M. K. GLEASON, M. D.,
*Registrar of Vital Statistics.*

---

### HEALTH DEPARTMENT.

### SMOKE INSPECTOR'S REPORT.

---

TO OSCAR C. DEWOLF, M. D.,
*Commissioner of Health.*

SIR:—Having been assigned, in addition to other duties, to the enforcement of Sections 1650, 1651, 1652 of the municipal code, known as the Smoke Ordinance, I respectfully submit the annexed report of my efforts to rid the city of this nuisance.

LOUIS MERKI,
*Inspector.*

SMOKE INSPECTOR'S REPORT FROM JULY 1882, TO DECEMBER 31, 1884.

| | |
|---|---|
| Notices served to abate smoke nuisance | 2,014 |
| Personal visits made and violations observed | 1,620 |
| Complaints made by communications | 264 |
| Complaints by persons in office | 130 |
| Abatements of the nuisance | 614 |
| Abatements not satisfactory | 89 |
| Suits commenced (but not prosecuted) nuisances abated | 268 |
| Suits prosecuted | 83 |
| Estimated number of boilers in use in city | 6,000 |

The Smoke Ordinance was passed by the City Council, April, 1881. Following is a copy of the Ordinance:

"SECTION 1650.—The emission of dense smoke from the smoke stack of any boat or locomotive, or from any chimney anywhere within the city, shall be deemed and is hereby declared to be a public nuisance: Provided, that chimneys of buildings used exclusively for private residences shall not be deemed within the provisions of this ordinance."

"SEC. 1651.—The owner or owners of any boat or locomotive engine, and the person or persons employed as engineer or otherwise, in the working of the engine or engines in said boat, or in operating such locomotive, and the proprietor, lessee and occupant of any building, who shall permit or allow dense smoke to issue or be emitted from the smoke stack of any such boat or locomotive, or the chimney of any building within the corporate limits, shall be deemed and held guilty of creating a nuisance, and shall for every such offense be fined in a sum not less than five dollars nor more than fifty dollars. Sections 1650, 1651, and 1652 shall take effect and be in force from and after May 1, 1881."

"SEC. 1652.—It shall be the duty of the Commissioner of Health and the Superintendent of Police to cause Sections 1650 and 1651 of this article to be enforced, and to make complaint against and cause to be prosecuted all persons violating the same."

Since the passage of the foregoing ordinance there have been over two thousand notices served to abate smoke nuisances. During the same period there have been seven hundred smoke-preventing appliances attached to furnaces, of which fully six hundred are now in successful operation ; all doubt as to the feasibility and legality of the ordinance have been dispelled, and business men generally are beginning to appreciate and approve the necessity of the ordinance. Eighty-three suits have been prosecuted against violators of the ordinance, but in all cases the nuisance was abated before the day set for trial. I have at all times endeavored to abate the nuisance rather than cause fines to be imposed. Great progress has been made within the last two years by the improvements of smoke-preventing devices and the invention of new ones; nowhere in the United States have they attained such a state of perfection in so short a time as in the city of Chicago. I have personally examined within two years over sixty different devices, but only six of them have proved successful, and each of the six now in successful operation differs in merits generally, but all abate the smoke nuisance. A great aid to any device is careful and judicious firing ; but this essential requirement is wanting in three boiler-rooms out of every four. First, only practical engineers should have charge of boilers and furnace-rooms. More fuel is wasted in many buildings than the salary of two engineers amounts to by unskilled firing. I find men in charge of boiler-rooms under buildings, where hundreds of people congregate daily, that do not know the difference between a direct and a return-flue boiler (thanks to the inventor of safety-valves). All they know is to fill the furnace full of coal and watch the steam gauge and peruse cheap works of fiction, often being so ingrossed in the contents thereof that the furnace is reduced to ashes, and the steam run down before firing; then overfeeding the furnace to get steam, these chimneys vomit 15 to 20 per cent. of the coal in the form of smoke into the air. A good, skilled fireman or engineer is half of any smoke consumer, unless it be an automatic one. Another cause of neglect in engine and furnace-rooms is the indifference of owners and proprietors to include daily the furnace-room in their rounds through their business offices. It may not only have a tendency to prevent the emission of dense smoke, but the coal bill may be reduced by these observations. The fuel should always be near the furnace, so that in firing the furnace doors are not kept open longer than absolutely necessary. It will also keep the fuel warmer and result in combustion more readily. Firing should be performed often and light, to insure perfect combustion, thereby getting the full benefit of the fuel consumed. With the devices now in use all grades of soft or bituminous coal can be successfully used for steaming purposes, and conform to the requirements of the smoke ordinance. Citizens generally should observe the following rules in selecting a device:

First. Its application to your furnace and cost.

Second. Its durability and fuel consumption.

Third. Its steaming capacity and effect on boiler and furnace.

Fourth. Does it prevent the emission of dense smoke?

The construction of a furnace, boiler-settings, grate-bars and draught are essential points, and it is with a degree of pleasure that I observe this improvement within the last year in new buildings erected, and those now in course of construction. An engine and furnace-room should be large and well lighted and ventilated. It should be made attractive instead of a dungeon under a sidewalk or in a sub-cellar, without light or air, where the heat is so intense and air so stifling that firemen are often obliged to leave their furnace-rooms for fresh air. This condition of affairs exists to an alarming extent in our business blocks and workshops, and is in many places ample excuse for neglect. As a whole, the results for the last two years are apparent to every citizen, and with continued enforcement of the ordinance the once great source of the smoke-nuisance will be a thing of the past.

Very respectfully,

LOUIS MERKI,

*Inspector.*

